# A rare case of periprosthetic joint infection of the hip due to Kocuria spp.

**DOI:** 10.1186/s12877-023-04286-2

**Published:** 2023-09-28

**Authors:** Nora Anais Koenemann, Fabian Sauerwald, Dierk Thimel, Edgar Mayr

**Affiliations:** Klinik Für Unfallchirurgie, Orthopädie, Plastische Und Handchirurgie; Universitätsklinikum Augsburg, Stenglinstr. 2, 86156 Augsburg, Germany

**Keywords:** Kocuria species, Periprosthetic joint infection, Total hip arthroplasty, Case report

## Abstract

**Background:**

*Kocuria spp*. are ubiquitous bacteria that have gained recent attention as potential infectious agents. The most common bacteria in PJI are *S. aureus* und *S. epidermidis*.

**Case presentation:**

We present the case of a 72-year-old woman who received total hip arthroplasty after a traumatic medial femoral neck fracture. Postoperatively, due to the clinical presentation of periprosthetic joint infection (PJI) revision surgery was performed twice. The microbiological tissue samples were positive for *Kocuria spp*. Initially, this was considered contamination and the patient was treated with various antibiotic regimens as well as prednisolone due to the differential diagnosis of pyoderma gangraenosum. However, a specialized histopathology lab performed further testing which substantiated the suspicion of a rare case of PJI due to *Kocuria spp*.

**Conclusions:**

To our knowledge, this is the first reported case of a PJI caused by *Kocuria spp*. Further clinical research is necessary to assess whether *Kocuria spp*. are an underestimated cause of PJI.

**Supplementary Information:**

The online version contains supplementary material available at 10.1186/s12877-023-04286-2.

## Background

Periprosthetic joint infections (PJI) are a therapeutically and economically relevant condition. Not only is there a high mortality [1–3], but reported costs in the United States already exceed 1.5 Billion USD with projected further increase of the economic burden [4, 5]. Despite many efforts to determine clear diagnostic criteria of PJI, such as the MSIS criteria [6, 7] and several other [8, 9] many cases remain challenging to diagnose. They are most commonly caused by *Staphylococcus aureus, streptococci* and *entrerococci* [10].

The *Kocuria species* is a Gram-positive cocci that was discovered in 1974 and has since been considered a non-pathogenic bacteria [11]. However, more recently there have been several case reports of infections due to *Kocuria spp*. These case reports have been mostly catheter-associated, or causative of uveitis, urinary tract infections and bacterial meningitis [12–14]. Although most *Kocuria* infections occur in immunocompromised patients, there have also been reports of *Kocuria* infections in immunocompetent patients [15–17].

One possible pitfall of PJI management has previously been the identification of bacteria [18]. According to the literature, this has improved significantly since the establishment of sonication as a routine diagnostic test [19, 20]. Histopathological examination of infected tissue has been discussed more in-depth in recent publications [21, 22]. In this report, we present a case of PJI due to *Kocuria spp*. where the diagnosis was made retrospectively by histopathological tissue specimen examination.

To our knowledge, this is the first reported case of PJI due to *Kocuria spp.*

## Case presentation

We report the case of a 72-year-old woman who presented to our emergency department (ED) after tripping over a suitcase.

Concerning the patient’s past medical history, she had migraines as well as suspected obstructive sleep apnea and depressive adjustment disorder. She had been treated for DVT following spinal surgery five years prior. She had no known allergies. She took no immunosuppressant medication.

In our ED we diagnosed a medial femoral neck fracture of the right hip. The patient received total hip arthroplasty within 24 h after presentation. There were no reported intra-operative complications. The patient was monitored on the intensive care unit (ICU) for two days postoperatively and was then transferred to the general ward. Initially, the surgical site showed no sign of infection. However, over the next days increased redness and persistent wound secretion developed. Furthermore, the laboratory values showed increased inflammatory markers with a C-reactive protein (CRP) value of 33,36 mg/dl (ref. range 0–0,5 mg/dl).

Due to these developments, PJI was suspected. Revision surgery was performed on day 7 after initial surgery with debridement, jet lavage and change of mobile parts. The intraoperative findings showed a sinus tract communicating with the prosthesis. 5 microbiological tissue samples were collected, which showed no bacterial growth within the first 48 h. Sonication was performed on the removed mobile parts and showed no bacterial growth within the first 48 h. No samples were sent for histopathological testing. Postoperatively, we began a calculated antibiotic regimen with ampicillin/sulbactam. However, the condition of the surgical site and the inflammatory markers continued to worsen so that another surgical revision was performed five days later. During the procedure, the hip prothesis was removed and a vancomycin-coated spacer was implanted. The intraoperative tissue samples and sonication were not positive for any bacteria. Post-operatively the patient was brought to our ICU where the antibiotic therapy was escalated to include vancomycin. A CT of the right leg revealed localized fluid retention in the subcutaneous tissue of the thigh (Image 2), compatible with soft tissue infection and an ultrasound-guided wound drain was placed.

Despite the escalated antibiotic regimen, the patient showed no signs of improvement. Further diagnostic testing (CT, ultrasound, blood cultures, urine cultures) was initiated but yielded no source of infection. At this point, seven days after the first revision surgery, the results of the sonication of the removed mobile parts revealed the presence of *Kocuria spp.*. In the consult by the microbiology department this was considered contamination.

As the findings were not unambiguous for PJI, the differential diagnosis of pyoderma gangrenosum was made and an empirical prednisolone therapy was initiated. Under the mixed regimen of antibiotics and prednisolone the infectious parameters normalized. However, the patient developed a macular pustular exanthema, which was considered an allergic reaction to vancomycin so that the antibiotic therapy was discontinued shortly. In consensus with the colleagues of the department of microbiology the antibiotic therapy was re-instated with linezolid and ciprofloxacin.

Due to the differential diagnosis of pyoderma gangraenosum we consulted the department of dermatology and rheumatology. Despite extensive testing, the diagnosis remained unclear but pyoderma was considered an unlikely diagnosis.

After 10 days on the ICU the patient was transferred to the regular ward. Over the following weeks, the infection constellation improved so that revision surgery was planned. Six weeks after removal of the prosthesis the reimplantation of a specialized Mutar® RS prosthesis with a tripolar, uncemented cup was performed. Intra-operatively gained tissue samples showed *Candida albicans* as well as *Staphylococcus epidermidis* but were considered contamination as they were only present in one out of five tissue samples. The antibiotic therapy with linezolid and ciprofloxacin was continued for two more weeks postoperatively. The prednisolone therapy was also continued and was eventually tapered out. Empirical short-term i.v. hydrocortisone showed no improvement. Due to the consistent uncertainty regarding the final diagnosis, empiric antimycotic therapy with fluconazole was added to the antibiotic regimen.

Results of the histopathological examination of the tissue samples from the final surgery where the Mutar® RS prosthesis was implanted, were not consistent with the diagnosis of pyoderma gangraenosum so that this differential was finally ruled out. Due to the inconclusive results of the microbiological testing of both intraoperative tissue samples as well as sonication results, we decided to perform further histopathological testing. The intraoperative tissue samples from the final revision surgery were sent to a specialized pathology laboratory (Prof. V. Krenn of Trier, Germany). Here, the histopathological examination revealed double-light bending foreign material, adipose tissue necrosis, foreign body reaction and heterotopic ossification. These finding were consistent with “bacterial low-grade infection, such as would be expected in an infection due to *Kocuria spp.*”. Hence, the final pathological diagnosis of the final revision surgery was a low-grade infection due to *Kocuria spp*. We therefore concluded that the initial bacterial agent causing the early-onset PJI was also due to *Kocuria spp.* The final diagnosis was therefore PJI due to *Kocuria spp..*

Due to these findings, the antimycotic therapy was discontinued and the antibiotic regimen was adapted to linezolid according to the initial antimicrobial sensitivity testing from the first surgery. It was continued intravenously for 2,5 weeks until discharge and then switched to oral application, recommended for another 10 weeks.

Throughout the entire hospital stay, the patient received physiotherapy. Finally, the patient was able to walk about 50 m with a high walking frame. The surgical site continued to show signs of slight redness, however there was no warming or secretion. The patient was discharged to a rehabilitative facility eleven weeks after her initial presentation.

In the follow-up presentations over the next three years, the patient reported increased difficulty with mobilization, mainly due to muscular weakness and stiffness in the hip. However, there were no signs of chronic or acute on chronic infection on the regularly performed radiographs, laboratory testing or physical examination.

## Discussion and conclusions

This case proved to be challenging both in terms of diagnosis and of treatment. Despite discrepancies in the clinical findings, PJI remained the most likely diagnosis throughout the course of treatment. The primary reason for this was the temporal connection between the first surgery and onset of symptoms.

In the literature, culture-negative PJI account for about 15% of PJI [23]. *Kocuria spp.* are usually considered to be non-pathogenic and cases of infections in immunocompetent patients have been reported but remain rare [11, 15, 16]. Furthermore, our patient had no known immunocompromising risk factors, neither by pre-existing condition nor by medication. This led our department of microbiology to initially assess the presence of *Kocuria* in the sonication as contamination and classify our case as that of a culture-negative PJI.

Our surgical approach to this case was consistent with our hospital’s SOP for PJI, which is largely based on the Proceedings of the International Consensus on Periprosthetic Joint Infection (Parvizi, 2013). We took a three-step approach first removing the mobile parts (equivalent to debridement, antibiotics, and implant retention, DAIR) and then explanting the prothesis due to persistent infection and placing a vancomycin coated-spacer. Due to the previously persistent infection and possible vancomycin allergy, we opted for a specialized hypoallergenic prosthesis for the final re-implantation.

Even though according to the MSIS Criteria for PJI [7] the initial intraoperative findings were positive for a major criteria, because of the negative cultures from the initial tissue samples and sonication as well as the lack of improvement under empirical antibiotic therapy, we considered other differentials. This led us to also consider pyoderma gangraeonsum as a differential diagnosis whilst continuing the treatment for PJI. However, despite improvement of laboratory values under prednisolone therapy, the dermatologist’s expert opinion was that pyoderma gangraenosum seemed unlikely. Finally, the histology results also showed findings inconsistent with pyoderma gangraenosum thus leading us to abandon this differential.

Throughout the course of treatment, we encountered several therapeutic challenges regarding antimicrobial therapy and the patient underwent several changes of the antibiotic regimen. This, again, was largely due to the absence of positive cultures as well as suspected development of an allergic reaction against vancomycin. The breakthrough in the case came after consultation of a specialized pathology laboratory. Histology has a low sensitivity for the detection of PJI but a high specificity and is especially useful in culture-negative cases [22]. The results of the histological examination from the tissue samples of the final surgery showed changes consistent with low-grade infection as could be expected due to *Kocuria* infection. We therefore concluded that *Kocuria spp*., which according to the sonication results was present at the time of the first revision surgery, was not a contamination after all. Histological examination of tissue samples is not standard protocol in our department in the treatment of early PJI and had not been collected during the initial revision surgery. Due to the low sensitivity of histology, it is questionable whether histological findings would have influenced the course of treatment. Nonetheless, it became retrospectively clear that *Kocuria spp*. was most likely the initial bacteria causing the early PJI in our patient. It remains unclear what patient-specific factors made her susceptible to infection with *Kocuria spp.*.

The diagnosis of PJI continues to be based on clinical findings without universal definite diagnostic criteria. Especially in the presence of culture-negative PJI, histopathological testing should be performed to confirm the working hypothesis. To our knowledge, this is the first reported case of 1.) *Kocuria spp*. as the bacteria causing PJI and 2.) *Kocuria spp*. PJI in an immunocompetent adult. We recommend considering typically non-pathogenic bacteria as the cause of infection when PJI is the most probable diagnosis and cultures would otherwise be considered negative. Further research is needed to identify factors that contribute to opportunistic PJI in seemingly immunocompetent individuals.

### Supplementary Information

**Additional file 1: Figure 1. **Development of Leucocyte values over the course of treatment, values in x10^9/L. **Figure 2.** Development of CRP values over course of treatment, values in mg/dl. 

## Data Availability

There is supplemental material to this paper which includes images and laboratory values.

## Abbreviations

CRP: C-Reactive Protein
CT: Computer Tomography
DVT: Deep Venous Thrombosis
DAIR: Debridement, Antibiotics and Implant Retention
ED: Emergency Department
ICU: Intensive Care Unit
MSIS: Musculoskeletal Infection Society
PJI: Periprosthetic Joint Infection
SOP: Standard Operating Procedures
Spp.: Species
