# Supplementary material for: A rare case of periprosthetic joint infection of the hip due to Kocuria spp
Source: BMC Geriatr. 2023 Sep 28;23:607. doi: 10.1186/s12877-023-04286-2 (PMC10536817; doi:10.1186/s12877-023-04286-2)
Supplement: Supplementary file 1 — Additional file 1: Figure 1. Development of Leucocyte values over the course of treatment, values in x10^9/L. Figure 2. Development of CRP values over course of treatment, values in mg/dl. [file 12877_2023_4286_MOESM1_ESM.docx]

**Title: A rare case of a periprosthetic joint infection of the hip due to Kocuria spp.**

Supplementary Material

Timeline:

Day 1 Fall, presentation to ED, diagnosis of medial femoral neck fracture

Day 2 Arthroplasty, total hip replacement, admission to ICU

Day 4 Transfer from ICU to normal ward

Day 7 Revision surgery: debridement, jet lavage, change of mobile parts

Day 12 Explantation of prosthesis, implantation of vancomycin-spacer

Day 14 Microbiological results: *Kocuria species*

Day 19 Transfer to ICU, insertion of ultrasound-guided drain into right thigh

Day 34 Transfer to normal ward

Day 60 Re-Implantation of hip prosthesis

Day 67 Pathology Results from Hospital Laboratory

Day 77 Pathology Results from Specialized Laboratory

Day 83 Discharge from hospital to rehabilitative center

Inflammatory Markers (CRP, WBC) over Course of Treatment

Figure 1: Development of Leucocyte values over the course of treatment, values in x10^9/L

Figure 2: Development of CRP values over course of treatment, values in mg/dl

Images


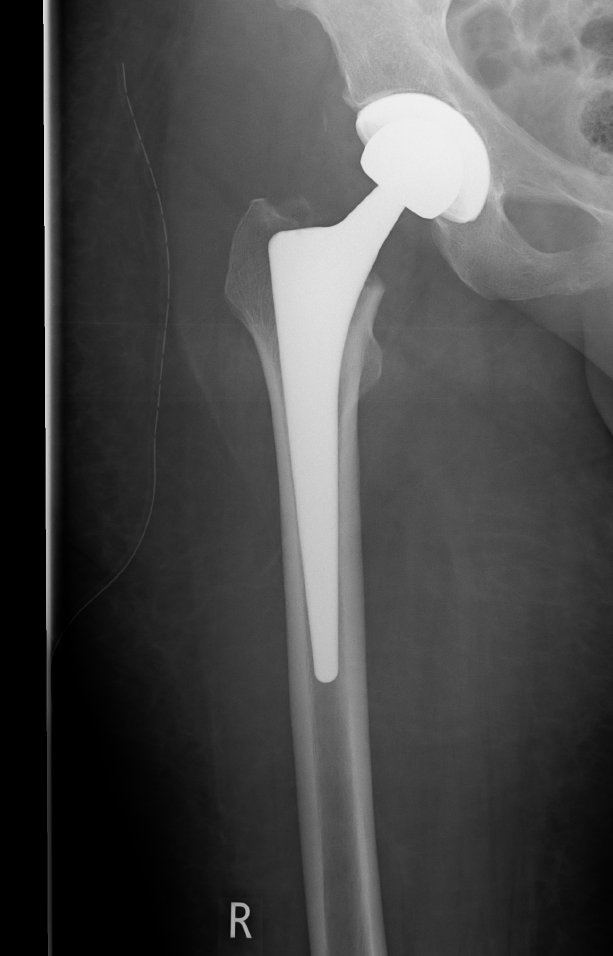


Image 1: Initial post-op x-ray after total hip arthroplasty


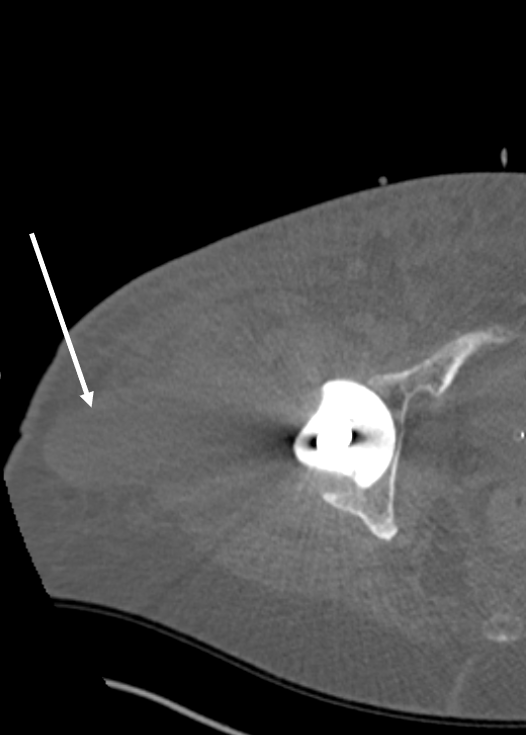


Image 2: Fluid retention (arrow) in the subcutaneous tissue in CT imaging


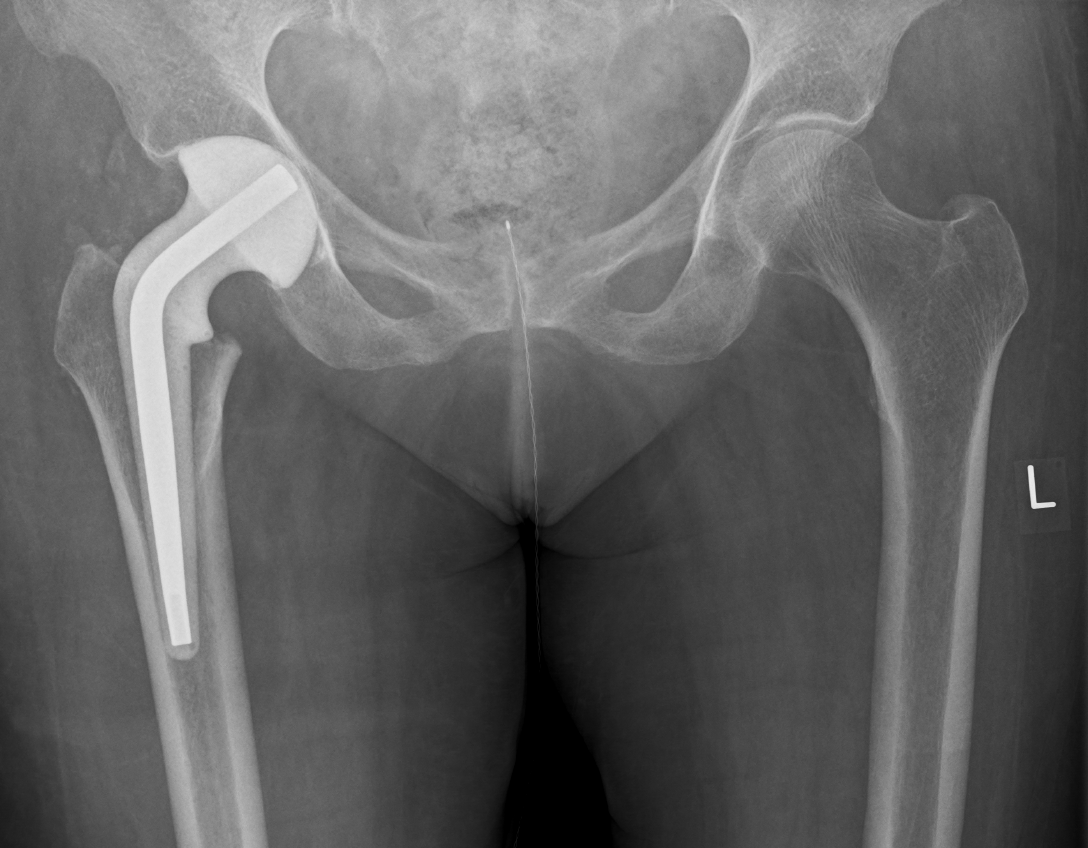


Image 3: Post-op x-ray of Vancomycin-coated Spacer


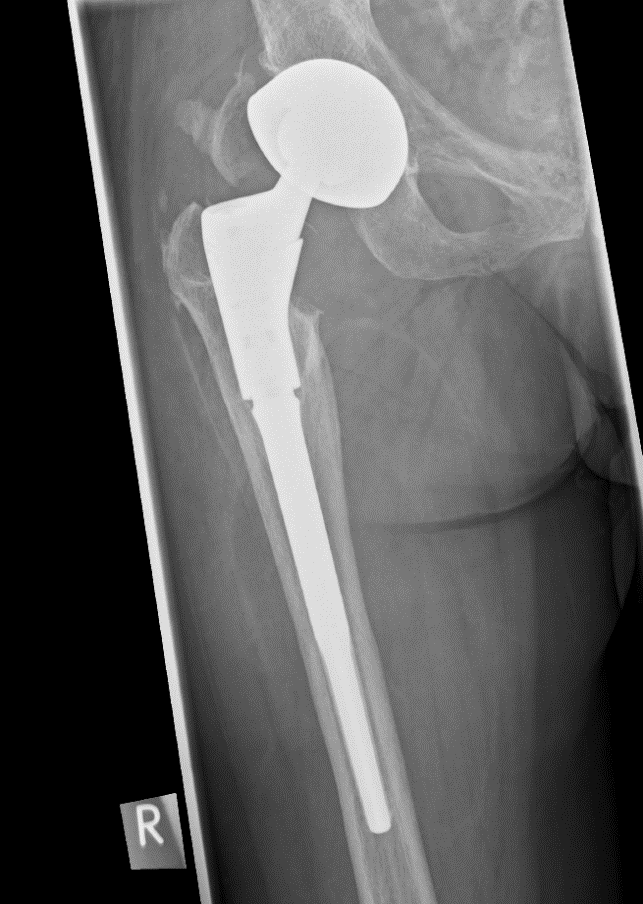


Image 4: Post-op x-ray of Mutar® RS prosthesis


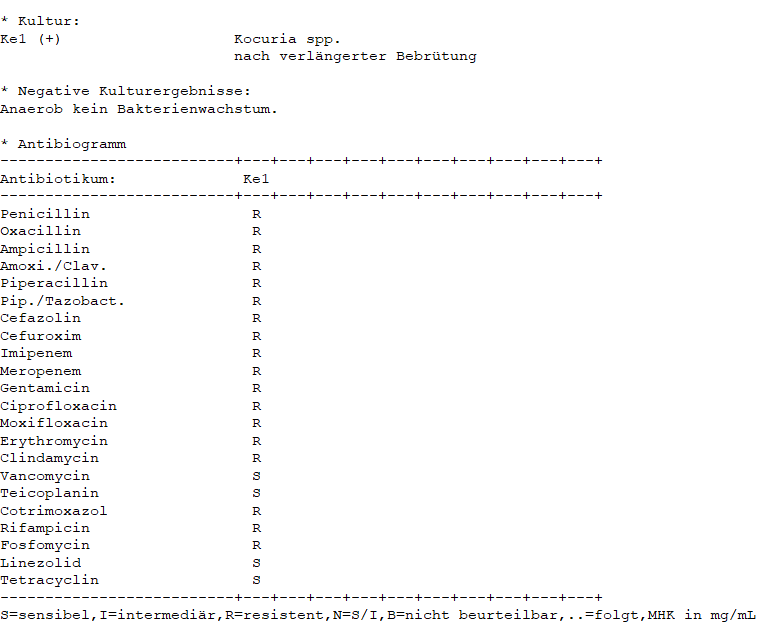


Image 5: Antibiotic suceptibility testing of Kocuria spp. isolated from sonication (in German)
